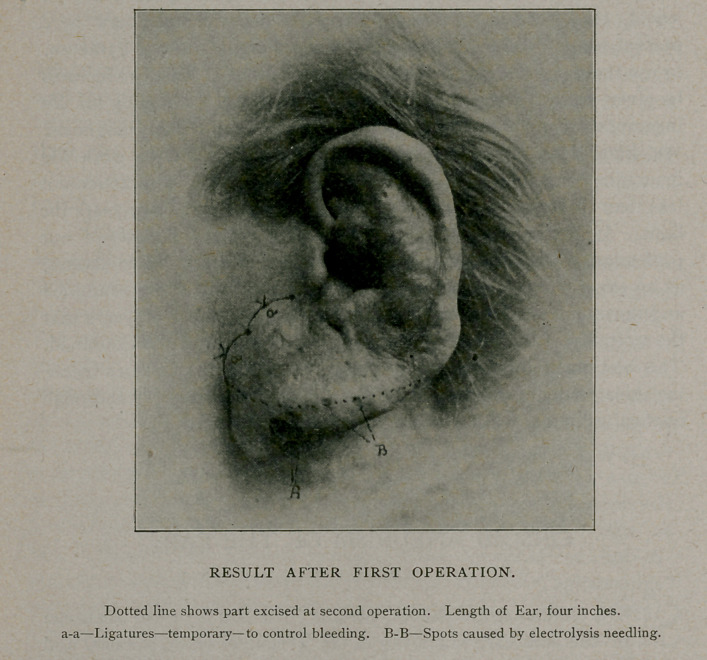# Cirsoid Aneurism of the Ear1Read at the thirty-third annual meeting of the Medical Association of Central New York, held at Rochester, N. Y., October 16, 1900.

**Published:** 1901-01

**Authors:** William S. Cheesman

**Affiliations:** Auburn, N. Y.


### CIRSOID ANEURISM OF THE EAR.[1]

By WILLIAM S. CHEESMAN, M. D., Auburn, N. Y.

SINCE childhood the patient, Mrs. C., has had a cirsoid aneurism of the left ear, which began as a simple telangiectasis, and gradually assumed the formidable appearance and character which I found at my first examination. The whole organ was then enlarged and protuberant, the lobe hanging low on the neck, and everywhere were visible throbbing and twisting arterial varices. The patient had had two children and was at this time four months pregnant. Some little time before, while going about her household duties, one of the

1. Read at the thirty-third annual meeting of the Medical Association of Central New York, held at Rochester, N. Y., October 16, 1900.

wicked-looking varices suddenly ruptured, shot a stream of blood across the room, and caused severe blood loss before it was controlled. The haunting fear of a recurrence of this accident was the prime cause of her seeking relief.

Pressure on the left common carotid completely controlled the pulsation in the ear. She was therefore advised to have the carotid ligated as a preliminary if not final measure, it being thought that this would remove the danger of sudden uncontrollable hemorrhage, and

RESULT AFTER FIRST OPERATION.

Dotted line shows part excised at second operation. Length of Ear, four inches.

a-a—Ligatures—temporary—to control bleeding. B-B—Spots caused by electrolysis needling.

that after her confinement further steps could be the more easily taken to reduce the size of the ear.

Deligation of the left common carotid was performed at the Auburn City Hospital, February 12, 1896, the vessel being tied with stout catgut above the tendon of the omohyoid muscle. Pulsation of the varices in the ear ceased at once with the tying of the ligature. The wound was closed with silkworm-gut and the patient rallied nicely from the anesthetic. Primary union ensued and no disagreeable effects referable to changes in the cerebral circulation were noted, save some transient headache and dizziness.

A photograph of the ear, taken before the operation, unfortunately scored a failure. The picture which I show you exhibits the ear after the first operation and after some attempts at needling with electrolysis needles, which proved unsatisfactory. It gives but a poor notion of the threatening aspect of the organ when I first saw it, when it resembled nothing so much as a squirming bundle of pulsating worms. It has shrunken much, and the venous sinuses are empty, only the organic scaffolding of the growth, the arterial and venous walls empty of blood, and the connective tissue, remaining. After operation the ear measured four inches in length, its fellow measuring two and one-half inches.

The patient was delivered of a healthy child during the summer of 1896, and returned to the hospital on December 1st for a plastic operation, to reduce the size of the enlarged lobule. A slight return of pulsation could now be felt in the ear, so, as a preliminary, two control ligatures were placed as shown in the photograph, and the lobule was shut off from the rest of the ear by long-bladed pressure forceps having rubber rings about the handles, the blades grasping the ear in its full width just below the anti-tragus. A piece of the lobule was then removed with scissors as shown by the dotted line in the illustration. The raw edges were whipped with large catgut and the ligatures and forceps removed. Considerable oozing ensued from the needle punctures, and a steady large flow in one place necessitated leaving on a hemostat for forty-eight hours.

I have since regretted that a larger mass was not at this time taken away. I counted on greater retraction than has occurred. The inflammatory reaction which ensued brought about some swelling of the ear and a thrombosis and consolidation of the venous sinuses, and some contraction has followed, but it has not been as great as I anticipated. On January 29, 1897, the ear measured three and a quarter, in June three and one-eighth, and in November three inches. But it has not greatly changed since that note and is still five-eighths inch longer than its fellow. All pulsation has ceased, but it remains rather a large member. I have advocated a further operation for the reduction of the remaining hypertrophy, but the patient is so well satisfied with her present safety and improved appearance that even the argument usually so convincing to her sex—namely, the cosmetic,—fails to move her.

## Figures and Tables

**Figure f1:**